# The effect of light during incubation and dark shelter enrichment on chick growth and behavior

**DOI:** 10.1016/j.psj.2025.106144

**Published:** 2025-11-21

**Authors:** Louisa Kosin, Emily O’Hara, Alex R. Johnston, Helen Brown, Lindsay J. Henderson, Simone L. Meddle

**Affiliations:** The Roslin Institute, The Royal (Dick) School of Veterinary Studies, Easter Bush, The University of Edinburgh, Midlothian EH25 9RG. UK

**Keywords:** Layer hen, Enrichment, Development, Early-life behavior, Incubation

## Abstract

Early-life conditions can influence chick development and welfare. In this study, we investigated the effect of light exposure during incubation, and a post-hatch dark shelter enrichment, on layer chick growth and behavior. White Leghorn chicken eggs were incubated under either full-spectrum white light (24L:0D) or darkness (0L:24D) in temperature-compensated photoperiodic boxes. After hatching, chicks were reared in either standard pens or pens with a dark shelter enrichment. The study was conducted using four cohorts of chicks, resulting in eight replicates per treatment combination. All cohorts were used to assess chick growth; three cohorts were used to measure behavioral time budgets and interaction with the dark shelter, and the final cohort was used to examine behavior while inside the dark shelter. Chick behavioral time budgets and dark shelter use was continuously recorded via video over four weeks and quantified. Hatching success was not affected by light during incubation but there were age- and sex-specific effects on body weight by four weeks, female chicks incubated under light, tended to be heavier than those incubated under dark conditions. The dark shelter enrichment reduced overall chick activity and foraging while increasing resting behavior, with effects dependent on incubation treatment and age. In response to enrichment, light-incubated chicks showed decreased activity and foraging, and increased resting, compared to dark-incubated chicks. In contrast, in standard pens, light-incubated chicks were more active and foraged more than dark-incubated chicks. Our novel findings show that light exposure during incubation shapes early-life behavior in layer chicks and modulates their responses to dark shelter enrichment. Further research is required to determine whether lighted incubation and post-hatch dark shelter enrichment improve layer pullet long-term welfare.

## Introduction

The environment experienced early in life can shape an individual’s physiology and behavior throughout its lifetime. Therefore, a broader understanding of how early-life environmental interventions influence behavior is essential for enhancing long-term animal welfare. In commercial poultry breeds, both incubation conditions and rearing environments can influence neurodevelopment and behavioral phenotypes (Chiandetti et al., 2017; De Haas et al., 2021). This suggests that optimizing pre- and post-hatch environments may enhance welfare outcomes in chickens reared for meat and egg production (Campbell et al., 2019). Under commercial standards, eggs are incubated under complete darkness, which contrasts with natural incubation patterns where embryos are regularly exposed to light, particularly towards the end of the incubation period (Archer and Mench, 2014). Studies have shown that light during incubation can improve poultry hatchability (in broilers: Shafey and Al-Mohsen, 2002; Tainika and Bayrakter, 2022; Tong et al., 2017), enhance growth (Rozenboim et al., 2004) and attenuate stress responses to the post-hatch environment (Özkan et al., 2012). In-ovo light exposure has also been reported to influence the development of the visual system and lateralization of the avian brain (Rogers, 1990), which is important for regulating diurnal rhythms (Wei et al., 2025), and affects visual learning and discrimination post-hatch (Rogers and Kaplan, 2019; Wichman et al., 2009). Despite these findings, the effects of incubation lighting on chicks’ behavioral engagement with post-hatch enrichments are little understood.

There is now growing attention regarding the effect of light during incubation on the potential to shape chick development and welfare outcomes. Previous studies suggest that light exposure during incubation can influence post-hatch behavior and stress susceptibility, at least in broilers, with chicks incubated in darkness showing increased indicators of fearfulness, such as higher vocalization rates, longer tonic immobility durations and higher circulating corticosterone concentrations, compared to chicks incubated under light (Archer and Mench, 2017, 2013). Fearfulness has been shown to be reduced following light during incubation in layer hens, but these effects are inconsistent across test paradigms and strains (Kliphuis et al., 2024; Manet et al., 2023a). Moreover, combined interventions in layer hens and pullets, such as incubation light and early-life prey provisioning, have not consistently reduced fearfulness, feather pecking, or improved post-vaccination recovery (Kliphuis et al., 2023) and visual discrimination (Vanden Hole et al., 2024).

Although numerous studies have investigated the effects of light during incubation on poultry welfare, synthesizing these findings is challenging due to methodological variability across studies such as differences in temperature, wavelength, light intensity, photoperiod and eggshell color (Riedel and Tzschentke, 2023). Additionally, most work to date has focused on endpoints related to fear, stress, and aggression, with little attention paid to how light during incubation affects general activity patterns or behavioral time budgets (but see: Drozdová et al., 2021; Güz et al., 2021; Özkan et al., 2022; Riedstra and Groothuis, 2004). Addressing this gap is critical for understanding how light during incubation can shape early-life behavior and for informing husbandry practices that promote chick welfare.

Environmental enrichment in early life, such as the provision of dark brooders, has emerged as a promising strategy to enhance chick welfare (Sirovnik and Riber, 2022). Dark brooders mimic aspects of maternal care by offering chicks a sheltered, dark space combined with supplemental heat, which has been shown to improve welfare (Edgar et al., 2016). As chicks mature, these enrichments promote greater three-dimensional use of the pen environment (Sirovnik and Riber, 2022). Welfare benefits include reduction in fearfulness (Forslind et al., 2025) and in injurious feather pecking, as well as the associated mortality from cannibalism (Gilani et al., 2012; Riber and Guzman, 2017). Most studies that have investigated the benefits of dark brooders have focused on their long-term effects on severe feather pecking, with less attention on how chicks interact with these enrichments in early life or how they influence behavioral patterns (Fält, 1978; Jensen et al., 2006). Dark brooders have also been shown to reduce locomotor activity, feather pecking, and fear responses during the first four weeks of life (Riber and Guzman, 2016), as well as increased behavioral synchrony (Riber et al., 2007) and improved quality of rest (Forslind et al., 2022; Martin-Cicera et al., 2024). Despite these reported benefits, dark brooders have not yet been widely adopted in commercial or laboratory settings (Sirovnik and Riber, 2022). As a potential alternative, simple and low-cost structures, such as unheated dark shelters (e.g., cardboard boxes), could provide similar opportunities for chicks to hide and rest as well as provide opportunities to perch. Yet, few studies have examined chick interactions with such shelters (but see Sherry, 1981) or their effects on behavior and welfare. This represents an important knowledge gap that would help determine whether this relatively simple enrichment could improve behavioral development and welfare outcomes in commercial poultry systems.

Refining both pre- and post-hatch conditions holds considerable potential to positively influence behavior and enhance welfare of laying hens. Therefore, in this study we investigated how light during incubation, and the presence of a dark shelter enrichment post-hatch, affected behavioral time budgets and growth in layer hen pullets in their first four weeks of life. We also assessed behavioral interactions with, and the use of a dark shelter enrichment. To explore the potential interactions between incubation conditions and postnatal enrichment, treatment interactions were also investigated.

## Material and methods

### Ethical statement

Light treatment during incubation, and the presence of a dark shelter, had no negative impacts on chick health or welfare. Parental stock and chicks were maintained in accordance with the UK Animals (Scientific Procedures) Act 1986. All experiments followed both the ARRIVE and NC3Rs guidelines and were carried out after ethical approval by the Veterinary Ethical Review Committee (VERC, Ref: 27.23), the Royal (Dick) School of Veterinary Studies, The University of Edinburgh.

### Experimental design

The experiments were conducted in four cohorts using a 2 × 2 factorial design which included a light vs. dark incubation treatment and the presence vs. absence of a dark shelter enrichment during rearing. This resulted in four treatment groups: 1) dark incubation without a dark shelter, 2) dark incubation with a dark shelter, 3) light incubation without a dark shelter and 4) light incubation with a dark shelter. For each cohort, chicks were housed in 8 pens across two rooms (4 pens per room), with treatments equally split across the rooms, resulting in two replicates per treatment group, per cohort. For the four experimental cohorts there was a total of 32 pens and 192 chicks (6 chicks per pen, 48 chicks per cohort). This provided eight replicates per treatment combination.

### Incubation conditions

White Leghorn chicken eggs from the National Avian Research Facility (NARF) at the Roslin Institute, UK, were set for incubation (*n* = 288). Eggs for all cohorts were obtained from the same parent breeding stock and were collected over three days prior to being set. For the four batches, each with 72 eggs per replicate, 36 eggs were incubated either under 24 h full-spectrum white light or 24 h darkness until hatching. Incubation was carried out in Brinsea Ovation 56 EX incubators (ID: AG47A), housed within two bespoke, temperature-compensated photoperiodic chambers (Tracksys Ltd.; 70 (l) x 80 (w) x 130 (h) cm). An illumination level of 100 lux at egg shell height inside the incubator (measured with a WT81B handheld digital lux meter) was achieved using two Exo Terra Natural Light Full Spectrum Daylight Bulbs (13 W, PT2190) positioned directly above the incubator (115 cm). During the 21-day incubation period, eggs and incubators were checked daily to maintain a stable temperature of 37.5 ± 0.2°C and relative humidity between 45 - 50 %, that was raised to 60 - 65 % on E18 for hatching. The incubators automatically turned the eggs for 90 ° every hour until E18. On E18, eggs were taken out of the egg carriers and placed on a thin foam mat on the bottom of the incubator for hatching. All chicks used in this study hatched within 24 h of day 21 of incubation. Post hatch, chicks were taken from the incubators, health checked, weighed and wing tagged (Datamars Livestock, Plastag A – 17 mm, color lime) for individual identification and transferred to pens in light and temperature-controlled rooms within the NARF. Chicks were not beak trimmed and surplus chicks were culled by cervical dislocation.

### Rearing conditions

**Fig. 1:**
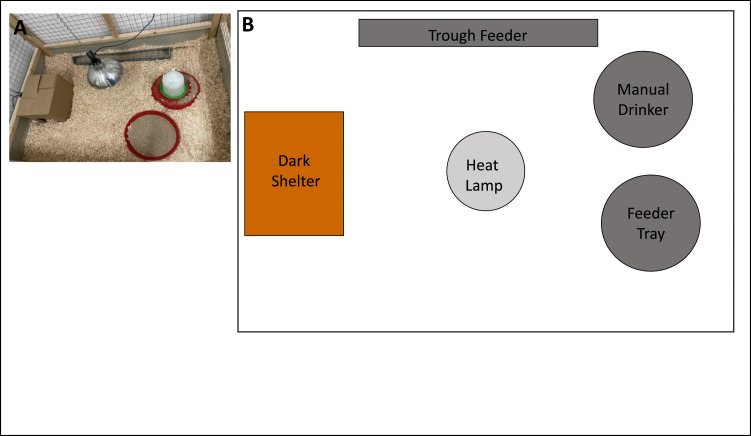
A) Representative image of the standard housing conditions, and B) a diagram of a pen layout equipped with 2 feeders, 1 drinker, a suspended heat lamp and a dark shelter enrichment (cardboard box).

Post-hatch, chicks were housed in eight pens across two light and temperature-controlled rooms (390 (l) x 400 (w) x 250 (h) cm) for four weeks. Four pens (175 (l) x 128 (w) x 195 (h) cm) were located in each room, with wooden frames and wire mash walls. Pens contained deep litter (wood shavings, Stevenson Brothers Avonbridge, UK), a circular red plastic chick starter feed tray (40 cm diameter), a galvanized steel chick trough feeder (50 (l) x 7 (w) x 8 (h) cm, a manual drinker (2l Chick Fount Plastic) and a heat lamp (30 cm diameter), all provided by Solway Feeders Ltd. (as shown in Fig. 1). All chicks had *ad libitum* access to a sterile rearing diet (Special Diet Services, HPS (P) 2 mm, Germany) and water, which was replenished every day. Two pens in each room contained a dark shelter enrichment (Fig. 1), consisting of a cardboard box (45 (l) x 30 (w) x 23 (h) cm; 6 % of pen floor area) with two openings at either end (14 (w) x 15 (h) cm). The dark shelter enrichment was large enough to house all chicks up to 4 weeks of age. Perches were not provided in pens, but chicks could perch on the manual drinker and trough feeder, or the dark shelter if provided. The chicks were visually isolated by an opaque section at the bottom of the pens (45 cm (h)), but not acoustically isolated from chicks in other pens. Chicks received Marek’s vaccinations within the first 3 days post-hatch, before behavioral observations commenced. Husbandry staff performed daily health checks. Initially, chicks were housed under 16L:8D then after 48 h, the day length was dropped to 15L:9D (lights on 06:30; lights off 21:30), with a 30 min. dawn and a dusk phase. Rooms were illuminated by Aquaforce Pro / AQFPRO S LED 4300 - 840, providing full-spectrum white light with 25 - 30 lux at feeder height in the pens and 0 lux recorded in the center floor area inside the dark shelter (assessed using handheld digital lux meter - WT81B). Rooms were maintained at a constant temperature of 27°C, with the temperature at floor level under the heat lamp being 33 - 35°C. Heat lamps remained in the pens for the four weeks of observations. Three weeks post-hatch, heat lamps were switched off and the room temperature was reduced to 25°C. Chick body weight (g) was taken at hatch, and then every 7 days post-hatch between 11:00 - 12:00, after the photoperiod was reduced to 15L:9D, outside of behavioral recording times.

### Behavioral observations

To record behavior, an infrared camera (GreenFeathersTM) was installed onto each of the pens (*n* = 8), 120 cm above the floor using 3D printed mounts. Cameras were angled towards the floor to record the full area of the pen (Fig. 1a), including underneath the heat lamp. Continuous video was recorded between 09:00 - 11:00, 13:00 - 14:00 and 17:00 - 19:00 each day for four weeks after hatch. Files were stored on a DVR (Hikvision 7200 Turbo HD Series DVR, 8 channel 6 TB), downloaded and backed up onto an external secure data server and hard drive once a week.

To quantify behavioral time budgets, chick behavior was assessed using scan sampling from the first three cohorts of chicks. This involved pausing the video every 5 min. for two 30 min. periods per day, resulting in 7 scans per observation. Scan sampling was performed three days per week (Saturday, Monday, and Wednesday) beginning at 09:00 and 13:00, resulting in a total of 4032 scans. The behavior of each chick in a pen was recorded in each scan, including their respective location: pen, perch, under heat lamp and inside the dark shelter. The behaviors recorded were: standing, resting, walking/running, foraging, consumptive behaviors (eating or drinking), and comfort behaviors. Behavioral definitions are outlined in an ethogram (see Table 1). Chick location in the pen was recorded as dark shelter if the chick was completely inside the shelter for more than 1 min. and in this case was assigned resting behavior. Chick behavior was quantified using BORIS behavioral analysis software v. 9.0.2 (Friard and Gamba, 2016). Two observers that were blind to the incubation treatment conducted the video observations, data from the video analysis were highly repeatable between (*n* = 8; *r* > 0.62, *P* < 0.01) and within observers (*n* = 8, *r* > 0.94 and *P* < 0.001) (Lessells and Boag, 1987). Observers were not blind to presence of a dark shelter or week of observation as this could not be concealed.

**Table 1:** Behavioral ethogram.

Behavioral Category	Behavior	Behavioral Elements	Active / Inactive
General Behaviors	RESTING	The chick is inactive on the floor of the pen or inside the dark shelter.	Inactive
	STANDING	The chick remains stationary without performing any other behavior.	Inactive
	WALKING / RUNNING	The chick takes two or more steps in any direction, including walking and running, with or without flapping its wings.	Active
	EATING / DRINKING	The chicks’ head is in the food tray or water drinker and or actively eating/ drinking.	Active
	FORAGING	The chick uses its feet to scratch at the ground kicking up or moving around litter and/or pecks at the substrate.	Active
Comfort Behaviors	DUST BATHING	The chick pecks and scratches at the litter followed by sitting ruffling wings in the litter moving dust through the feathers. Chicks may also rub or roll about in the litter.	Active
	PREENING / RUFFLING	The chick squats down onto the substrate and follows an organized sequence of behavior patterns, moving head and beak through the feathers, the chick may be standing or lying down. This also includes puffing up feathers or shaking.	Active
	WING FLAP / WING STRETCH	The chick flaps or extends its wings but remains stationary. The chick uses foot to stretch out its wing.	Active
Location	UNDER HEAT LAMP	The chick is directly under the heat lamp.	NA
	PEN	The chick is anywhere else in the pen, on the floor of the pen.	NA
	UNDER / IN DARK SHELTER	The chick is fully inside the dark shelter for more than 1 min.	NA
	PERCH	The chick is perched on top of the dark shelter, drinker, side of the pen or feeder.	NA

### Behavior inside the dark shelter

To observe the behavior inside the dark shelter, cameras were placed inside the dark shelters for the fourth cohort of chicks only (4 pens, 6 chicks per pen). Infrared nest box cameras (*n* = 4, GreenFeathersTM) were installed at the junction of roof and back wall of each dark shelter, with cables attached to the side of the wire wall of the pen. This provided a full view of the floor area and the two openings. Chicks rarely used the enrichment during the first week of life, therefore continuous observation methods were used to assess the behavior during that time, each shelter was observed for 90 min. at 09:30 and 13:00, on two days (180 min. total). To measure the frequency of behaviors in the dark shelters for weeks 2 - 4, each shelter was observed on two days per week for 60 min. at 09:30 and 13:00 (120 min. total), and scan sampling was performed every 5 min. This resulted in 14 scans per hour, totaling 672 scans for weeks 2 - 4. Behaviors were assigned using the previously established ethogram (Table 1) and quantified using BORIS behavioral analysis software v. 8. 25 (Friard and Gamba, 2016). To assess the time spent performing each behavior inside the dark shelter, each behavior duration was quantified from samples collected over three weeks of observations, totaling 12 instances each of resting, standing, walking or running, foraging, and comfort behaviors.

At the end of the experiment for each cohort, chicks were weighed to the nearest 0.1 g and culled by cervical dislocation. Chicks were sexed post-mortem based on reproductive organs, and plumage condition was assessed for any evidence of feather pecking, but none was observed.

### Statistical analysis

All statistical analyses were performed in SAS Software Version 9.4 (2024).

***Chick body weight.*** A linear mixed model (LMM) was fitted to the weekly live chick body weights (log transformed) from ages 0-4 weeks for the four cohorts. Fixed effects were incubation treatment (Light, Dark), enrichment (Dark Shelter, Control), sex, week, pen, cohort and their interactions. Random effects included were pen and its interactions with week and cohort. An unstructured covariance matrix was applied for repeated measures, accounting for any correlations across time points and incorporating Chick ID into the model to avoid pseudoreplication. Post hoc tests for the interaction between enrichment and incubation treatment for each week are based on P values from approximate t-tests for the contrasts between incubation treatment for each week of the study.

***Behavioral time budgets and location.*** The behaviors statistically analyzed from the original ethogram included proportion active (included all active behaviors; walking / running, foraging, consumptive behaviors (eating / drinking) and comfort behaviors). Individual behaviors analyzed were the most commonly observed; proportion foraging, resting and consumptive behaviors (eating / drinking). The proportion of chicks performing each behavior, and the proportion of chicks in each location, was taken over the 7 scans per session per pen. For each cohort (*n* = 3) that resulted in 24 records per pen for each behavior class and location (2 sessions per day, 3 days per week, over 4 weeks).

To analyze behavior and space use in pens, LMMs were fitted to behavioral and location proportions from each session. Fixed effects were incubation treatment (Light, Dark), enrichment (Dark Shelter, Control), pen sex-ratio, week, time of day, day, room, cohort and their interactions. Random effects included were pen and its interactions with week and cohort. Post hoc tests for the interaction between enrichment and incubation treatment for each week are based on P values from approximate t-tests for the contrasts between enrichment and incubation treatment for each week of the study.

To control for multiple testing, we have applied a Bonferroni Correction to adjust the P value significance threshold, based on 0.05/number of tests, for individual behaviors (foraging, resting and consumptive behaviors) 0.05/3 and location (pen, dark shelter, heat lamp, perch) 0.05/4. Therefore, effects were only interpreted as significant when *P* ≤ 0.02 and *P* ≤ 0.01 for behavior and location respectively.

Some chicks (*n* = 4) were excluded from the body weight analyses as they died or were culled prior to the end of the observations for health reasons. In cohort 2, one chick developed a leg injury and was culled, and one other chick did not thrive and died in the first few days after hatch. In cohort 4, two chicks did not thrive and died within the first few days after hatch. Pens with 5 chicks were included in behavioral analyses, as behaviors were calculated as a proportion. Video observations for one pen in cohort 2 were lost due to camera failure (all observations for Week 2 and three observations in Week 3).

## Results

### Chick body weight and hatching success

Incubation treatment did not affect hatching success (Hatch rate mean ± SE: Dark (82.6 % ± 4.3); Light (79.2 % ± 2.9); t-test_(3)_ = 0.50, *P* = 0.65). There was a significant effect of chick age (week) and sex upon chick body weight, with chicks gaining weight over the four weeks (F_(3,12)_ = 22008.8, *P* < 0.001) and male chicks were significantly heavier (F_(1,160)_ = 38.0, *P* < 0.001). In the full model there were significant two-way interactions, Week * Sex (F_(3,477)_ = 58.7, *P* < 0.001) and Week * Incubation Treatment (F_(3,12)_ = 3.84, *P* = 0.04). To investigate this further, models were run separately for the sexes. For female chicks, there was a significant Week * Incubation Treatment effect (F_(3,80)_ = 5.1, *P* < 0.01, Table 2). Post hoc tests showed a near significant trend that female chicks incubated under light conditions were heavier compared to those incubated under dark by week 4 (*P* = 0.06, Table 2). For both male and female chicks, dark shelter enrichment did not affect chick body weight (Male: F_(1,17)_ = 0.08, *P* = 0.78, Female: F_(1,13)_ = 0.02, *P* = 0.88).

**Table 2:** Live chick body weight (± S.E.M) at hatch (g), and the end of weeks 1, 2, 3 and 4 (cull) of male and female chicks incubated under light or dark conditions.

Sex	N	Incubation Treatment	Hatch	Week 1	Week 2	Week 3	Week 4
M	58	Light	40.0 ± 0.8	81.1 ± 1.6	141.4 ± 3.0	221.8 ± 5.7	322.9 ± 8.5
M	38	Dark	40.1 ± 0.9	83.0 ± 2.4	144.8 ± 3.8	226.1 ± 5.6	322.4 ± 10.8
F	36	Light	39.3 ± 0.8	79.7 ± 1.7	135.8 ± 3.2	205.0 ± 5.1	290.3 ± 7.2
F	56	Dark	39.4 ± 0.8	80.0 ± 1.6	135.3 ± 2.6	203.6 ± 3.9	281.2 ± 6.0

### Behavioral time budgets

**Fig. 2:**
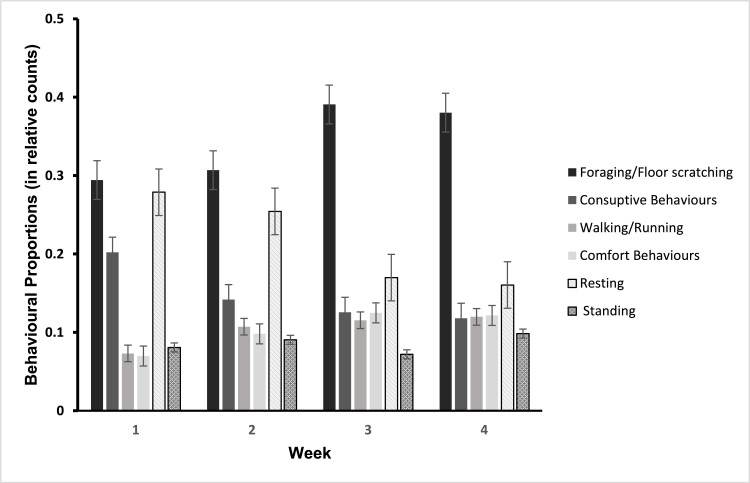
Proportions of chick behaviors, expressed as relative counts of total observed behaviors over the 4 weeks of the experiment (n = 24 pens, foraging, consumptive behavior (eating and drinking), walking / running, comfort behaviors, resting and standing). Graphs display mean ±SE.

As chicks grew older, they became more active, showing a higher relative incidence of foraging and a lower incidence of resting and consumptive behaviors (Fig. 2). The effect of the dark shelter on chick behavior depended on incubation treatment and chick age, as indicated by significant three-way interactions between Incubation Treatment * Enrichment * Week, for total activity (F_(3,282)_ = 4.31, *P* = 0.005), foraging (F_(3,282)_ = 3.34, *P* = 0.02), and a near significant trend for resting (F_(3,282)_ = 2.60, *P* = 0.05).

**Fig. 3:**
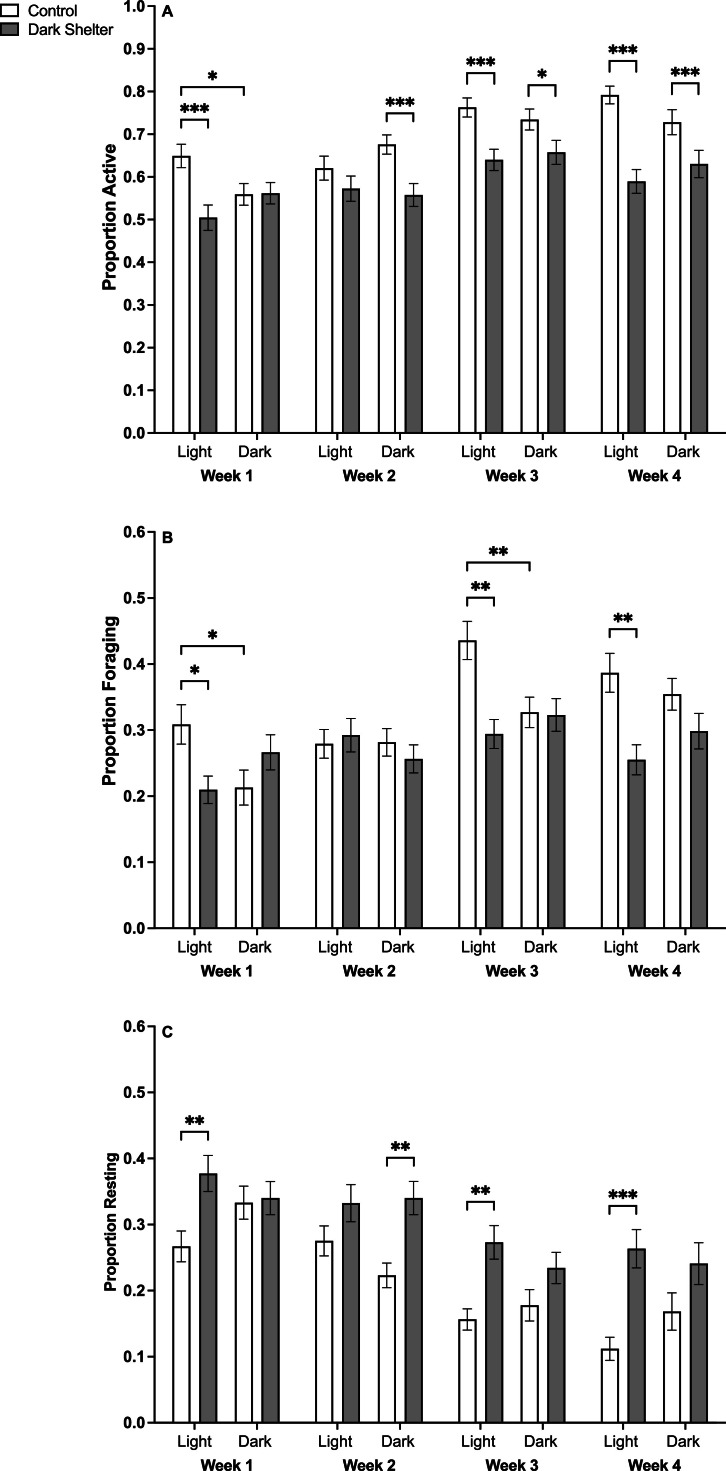
Proportion of A) total active behaviors, B) foraging and C) resting, for chicks over four weeks for each treatment. Proportions were calculated per pen (n = 24); * = P < 0.03; ** = P < 0.01; *** = P < 0.001. Graphs display mean ±SE.

Overall, chicks housed with a dark shelter enrichment were less active than chicks with no enrichment (Fig. 3a, F_(1,282)_ = 32.6, *P* = 0.001). Post hoc tests showed that in the first week after hatching, chicks incubated under light and housed with enrichment were less active than those in control pens (*P* < 0.001), this difference was not observed in chicks incubated under dark conditions (*P* = 0.83). Also in the first week, there was a trend for chicks incubated under light to be more active than those incubated under dark conditions in control pens (*P* = 0.03). By weeks 3 (*P* < 0.001) and 4 (*P* < 0.001), chicks in pens with a dark shelter were significantly less active than controls, in both incubation treatments. In week 2, this effect was observed only in dark-incubated chicks (*P* < 0.001).

For both incubation treatments, chicks housed with dark shelter enrichment foraged less than those in control pens by weeks 3 and 4 (Fig. 3b, F_(1,282)_ = 8.47, *P* = 0.004). Post hoc tests revealed that this reduction in foraging in enriched pens was significant in weeks 1 (*P* = 0.02), 3 (*P* = 0.001), and 4 (*P* = 0.001), for chicks incubated under light conditions only. No significant difference was observed for chicks incubated in the dark (*P* > 0.12). Additionally, in control pens, light-incubated chicks foraged significantly more than dark-incubated chicks in weeks 1 (*P* = 0.05) and 3 (*P* = 0.005).

Chicks in pens with the dark shelter enrichment rested more than those in control pens, for both incubation treatments (Fig. 3c, F_(1,282)_ = 20.8, *P* < 0.001). Post hoc tests revealed that this effect was significant for chicks incubated under light in weeks 1 (*P* = 0.006), 3 (*P* = 0.004), and 4 (*P* < 0.001), while only significant in week 2 for chicks incubated under dark conditions (*P* = 0.002).

The presence of the dark shelter also resulted in a reduction in consumptive behaviors (F_(1,282)_ = 6.22, *P* = 0.01). The incidence of consumptive behaviors also declined with chick age (Week: F_(3,59)_ = 2.60, *P* < 0.001), and there was a near significant trend that light-incubated chicks showing fewer consumptive behaviors than dark-incubated chicks (F_(1,282)_ = 3.82, *P* = 0.05). All interactions were non-significant at *P* > 0.25.

### Behavioral engagement with the dark shelter enrichment

**Fig. 4:**
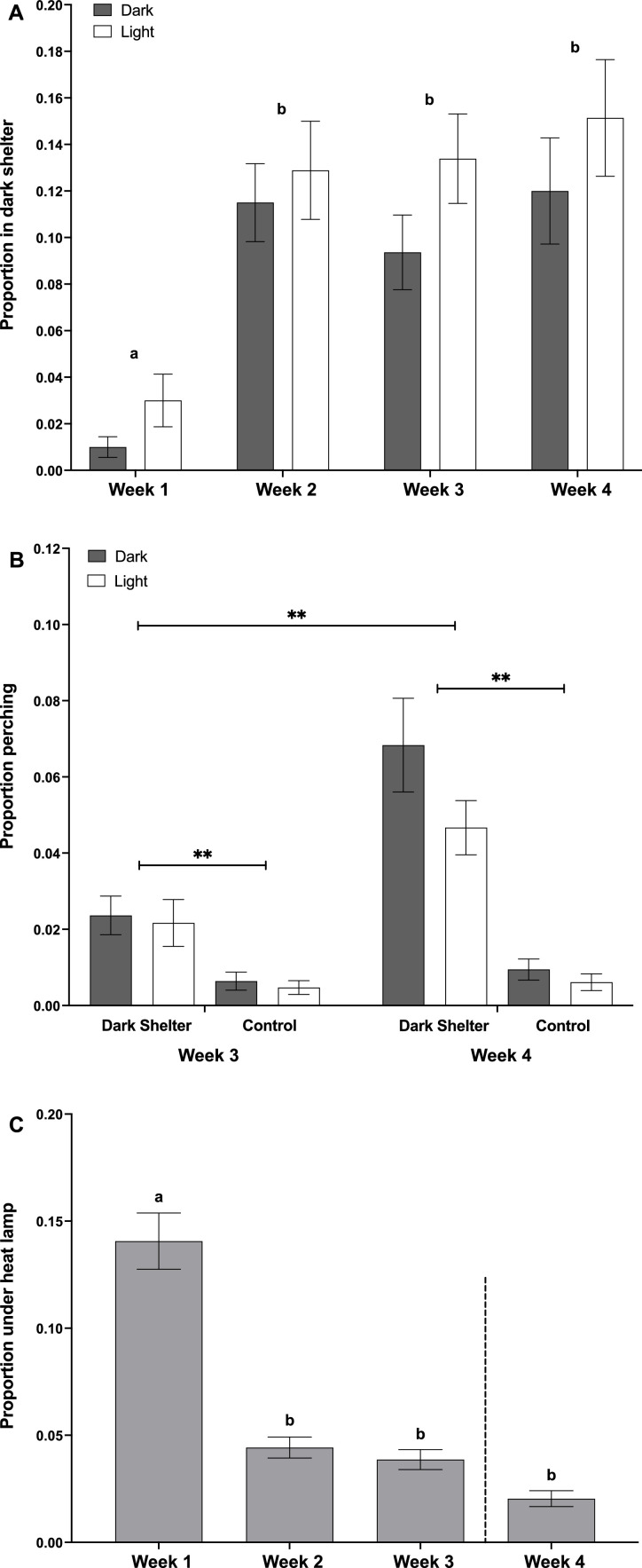
A) Proportion of chicks observed in the dark shelter during each week of the study, for light and dark incubation treatments, in pens with dark shelter enrichment only (n = 12). B) Proportion of chicks perching during weeks 3 and 4 in control and dark shelter enrichment pens, for both light and dark incubated chicks (n = 24) and C) proportion of chicks observed under the heat lamp each week across all treatments (n = 24). The dashed line indicates when the heat lamps were switched off. For Figs. A and B, a and b denote significant differences between weeks at P < 0.01. ** denotes significant differences at P < 0.01. Graphs display mean ±SE.

Chick use of the dark shelters increased with age, rising from 2 % of scan observations in the first week to 12 % during weeks 2 - 4 (Fig. 4a; Week: F_(3,29)_ = 19.81, *P* < 0.001). Incubation treatment had no effect on shelter use (Fig. 4a; F_(1,138)_ = 0.21, *P* = 0.65). Perching behavior was significantly affected by the presence of the dark shelter. In weeks 3 and 4, chicks provided with a dark shelter perched more frequently than chicks in control pens (Fig. 4b; Enrichment * Week: F_(3,282)_ = 17.8, *P* < 0.001), with a significantly higher proportion perching in week 4 compared to week 3 within the enriched pens (*P* < 0.01). Perching behavior was unaffected by incubation treatment (Fig. 4b; F_(1,282)_ = 0.05, *P* = 0.83). Heat lamp use was highest in week 1 and declined over time (Fig. 4c; F_(3,59)_ = 35.4, *P* < 0.001), with no significant effect of either enrichment (F_(1,282)_ = 0.5, *P* = 0.48) or incubation treatment (F_(1,282)_ = 0.01, *P* = 0.94).

Chicks were mostly inactive while inside the dark shelter enrichment, with 63 % of observations showing them either resting or standing (Table 3). Resting was the most prolonged behavior, with a mean duration of 336 seconds. In contrast, no other observed behavior lasted longer than 1 min.

**Table 3:** The frequency of, and duration spent performing the different behavioral classes inside of the dark shelter enrichment, from four weeks of observations (pens n = 4, one cohort).

Behavior inside dark shelter	Count		Duration (s)			
	Total	%	mean	SEM	min	max
Resting	150	0.44	336.0	71.7	34	817
Standing	66	0.19	7.5	0.8	3	12
Walking or running	53	0.16	4.5	1.0	1	13
Foraging	37	0.11	18.3	4.4	3	55
Comfort behaviors	35	0.10	11.9	3.0	2	42

## Discussion

This study evaluated the effects of low-intensity full-spectrum white light exposure during incubation and the provision of a dark shelter enrichment on layer chick growth and behavior. A modest increase in body weight was observed in light incubated female chicks at four weeks of age, but post-hatch enrichment was not found to affect body weight. Throughout the four-week study, chicks actively engaged with the dark shelter, using it increasingly over time, primarily as a place to rest. Notably, chicks incubated under light showed increased engagement with the dark shelter compared to those incubated under darkness.

### Impact of incubation treatment and dark shelter upon body weight

Our results indicate that body weight at four weeks of age was higher in female chicks incubated under light. This finding aligns with previous studies reporting post-hatch increases in body weight following lighted incubation (Bai et al., 2016; Güz et al., 2021; Wang et al., 2021). However, the impact of light during incubation upon pullet growth is mixed and appears to be influenced by genetic background (Hannah et al., 2020; Manet et al., 2023b), and factors such as the duration (photoperiod), wavelength, and intensity of light exposure during incubation (Drozdova et al., 2021; Riaz et al., 2021; Zhang et al., 2012). Importantly, sex-specific growth responses to early-life environmental cues are particularly relevant for the layer industry, where only female chicks are reared.

Although female light incubated chicks tended to be heavier by four weeks of age, behavioral observations suggested they spent less time performing consumptive behaviors (eating and drinking) and more time foraging relative to dark-incubated chicks. One possible explanation is that light incubated chicks were more efficient at converting food into body mass, or that increased foraging activity allowed them to locate and select more food items out with their food dish. Thus, these patterns may not be contradictory but rather reflect differences in behavioral strategies and energetic efficiency. However, further research is required to test this hypothesis, and given the marginal significance of these findings, they should be interpreted with caution, especially when relating them to industrial pullet rearing conditions.

While incubation conditions can play a role in early growth, they are not the sole determinants. Environmental enrichment following hatch has also been shown to influence growth trajectories (Jones, 2001). In the present study, the provision of a dark shelter post-hatch did not affect body weight. This aligns with a study by Forslind et al. (2025), where dark brooder presence during rearing had no effect on body weight or feed-conversion ratio. Nevertheless, the dark shelter significantly affected behavioral patterns, particularly in light incubated chicks. This suggests that incubation treatment and post-hatch enrichment could contribute to long-term effects on growth, that could emerge in later life stages.

No effects of the light treatment on hatchability, hatch weight, or early chick mortality were observed as a low light intensity was selected for incubation in this study. This adds to a growing body of evidence suggesting that light during incubation has a limited impact on hatching success (Riedel and Tzschentke, 2023). However, the light intensity matters, as high-intensity lighting reduces hatchability (Shafey and Al-Mohsen, 2002) and increases embryonic mortality (Shafey et al., 2005).

### Impact of incubation treatment and dark shelter upon behavior

The discovery that embryonic photostimulation determined behavioral interactions with the post-hatch enrichment is novel, and potentially provides an opportunity to positively influence neurodevelopment, chick behavior and improve poultry welfare. Here we applied 24 h of low-intensity continuous light throughout incubation to provide a consistent light stimulus without choosing a specific photoperiod, wavelength, or timing during development. This approach was chosen because previous studies report mixed behavioral outcomes depending on these parameters, and our aim was to isolate the effect of light exposure during incubation on post-hatch behavioral responses to enrichment. Chicks housed with a dark shelter enrichment were less active, foraged less, and rested more, and these effects were found earlier in life, and were more significant in light incubated chicks. In addition, light incubated chicks in standard pens were more active and foraged more than dark incubated chicks. Light during incubation may have affected neurodevelopment resulting in more adaptable and less novelty adverse chicks (Manet et al., 2025). In particular, there are reports that photostimulation during incubation results in higher levels of cerebral lateralization in chicks, which can ultimately influence the behavior and interaction with resources and novel objects (Chiandetti and Vallortigara, 2019). Further investigation is required to evaluate the long-term welfare implications of lighted incubation and post-hatch dark shelter enrichment in layer hens.

Dark shelter provision increased resting behavior, consistent with previous findings (Sirovnik and Riber, 2022). This effect may be attributed to improved rest quality provided by the shelter, which has been shown to support more synchronized, prolonged, and less disturbed resting bouts (Forslind et al., 2022; Martin-Cicera et al., 2024; Riber et al., 2007; Riber and Guzman, 2016). Notably, the impact of the dark shelter upon behavior was evident as early as the first week post-hatch, despite limited instances of chicks resting inside the shelter itself. Interestingly, chicks were also observed resting along the outer walls of the shelter. Given the positive thigmotactic tendencies of chicks (Wright et al., 2020), the shelter may have enhanced resting behavior by increasing the available perimeter within the pen, thereby offering additional support for rest. These findings underscore the value of providing dark shelter from hatch, reinforcing previous evidence that early-life environmental enrichment can positively influence chick behavior and promote welfare outcomes (Jensen et al., 2006). However, these results should be interpreted with caution when applying the findings to commercial production systems, due to the low stocking densities used.

The overall prevalence of behaviors we observed aligned with previous research, specifically that foraging was the most frequently observed behavior (Dawkins, 1989). This supports the reliability of our methodological approach for capturing commonly expressed behaviors. However, comfort behaviors such as preening, dust bathing, and wing stretching were observed in less than 10 % of scans, indicating that scan sampling may be insufficient for accurately quantifying low-frequency or short-duration behaviors (Tacha et al., 1985; Wilder et al., 2021). Future studies incorporating measures of behavior indicative of positive affect would be valuable to assess whether the increased use of the dark shelter by light-incubated chicks translates into enhanced welfare outcomes (Mellor and Beausoleil, 2015).

### Chick engagement with the dark shelter

Our study showed that chicks actively use the dark shelter enrichment in the first weeks of life, predominately for resting. The use of dark shelter enrichment increased over time, consistent with previous research that has shown that environmental exploration also increases with age (Pritchett-Corning, 2020). A dark shelter enrichment, without a heat source, was specifically chosen to control for heat-seeking behavior as a confounding variable and increase the feasibility of implementation in commercial production systems. In this study, chicks preferred to spend time under the heat lamp during the first week of life, rather than in the dark shelter, reflecting their inability to thermoregulate independently, a capacity that does not develop until about 12–14 days after hatching (Romijn, 1954). Our results also showed, that the perching behavior of chicks increases with age, especially in pens with the dark shelter. The dark shelter created a multipurpose enrichment, by simultaneously providing both shelter for resting and perching opportunities. Providing environmental enrichment can lead to decreased floor space (Newberry, 1995), therefore optimizing space use through multipurpose enrichments could be an efficient solution. Chicks housed with dark shelters perched more frequently by three and four weeks of age, highlighting how this multipurpose enrichment supports varied behaviors during early chick development. Rearing chicks with perches has several welfare benefits including improved bone strength (Anderson et al., 2024) and spatial cognition (Tahamtani et al., 2015). The dark shelters also provided the chicks with a space to rest, validating previous findings on chick behavior in response to dark brooders (Riber and Guzman, 2016). Their continued use of the dark shelter to rest up to four weeks of age, also suggests the benefits persist after the initial brooding period. Providing a dark space (0 lux inside the shelter) might have encouraged primarily inactive behaviors, for example broiler and layer chicks prefer higher light intensities for active behaviors like feeding, and lower intensities for inactive behaviors like resting until six weeks of age (Davis et al., 1999).

The chicks also used the dark shelter enrichment for other behaviors including running through the shelter and resting against its outside walls. The enrichment increased the “edge” provided in the pens, which has been shown to decrease aggression and disturbance during rest (Cornetto and Estevez, 2001), highlighting how three-dimensional enrichments can offer diverse stimuli. Our findings indicate that dark shelter enrichment is valued by young chicks, suggesting that housing designed to meet such behavioral preferences can promote better welfare outcomes throughout development. Follow-up studies are necessary to assess the relative benefits of unheated dark shelters and dark brooders and to determine if results persist with higher stocking densities, to form decisions regarding implementation in commercial practice.

## Conclusions

This study provides the first evidence that light during incubation can modify post-hatch interaction with a dark shelter enrichment. It validates the dark shelter as a suitable enrichment for chicks in early life, and highlights its multipurpose usage by the chicks as they grow. Future studies conducted under commercial conditions with higher stocking densities are needed to determine whether our findings persist in real-world settings. This would help identify new opportunities for the poultry industry to enhance chick welfare through precise manipulation of early-life environments, including incubation lighting and dark-shelter enrichment.

## CRediT authorship contribution statement

**Louisa Kosin:** Writing – original draft, Methodology, Investigation, Formal analysis. **Emily O’Hara:** Investigation. **Alex R. Johnston:** Writing – review & editing, Investigation. **Helen Brown:** Writing – review & editing, Formal analysis. **Lindsay J. Henderson:** Writing – review & editing, Supervision, Project administration, Methodology, Investigation, Funding acquisition, Formal analysis, Data curation, Conceptualization. **Simone L. Meddle:** Writing – review & editing, Supervision, Methodology, Investigation, Funding acquisition, Conceptualization.

## Disclosures

The authors declare that they have no known competing financial interests or personal relationships that could have appeared to influence the work reported in this paper.
